# Where the rubber meets the road — An integrative review of programmatic assessment in health care professions education

**DOI:** 10.1007/s40037-020-00625-w

**Published:** 2020-10-21

**Authors:** Suzanne Schut, Lauren A. Maggio, Sylvia Heeneman, Jan van Tartwijk, Cees van der Vleuten, Erik Driessen

**Affiliations:** 1 School of Health Professions Education, Department of Educational Development and Research, Maastricht University, Maastricht, The Netherlands; 2 Department of Medicine, Uniformed Services, University of the Health Sciences, Bethesda, MD USA; 3 School of Health Professions Education, Department of Pathology, Cardiovascular Research Institute Maastricht, Maastricht University, Maastricht, The Netherlands; 4 Department of Education, Utrecht University, Utrecht, The Netherlands

**Keywords:** Programmatic Assessment, Knowledge synthesis, Health Care Professions Education

## Abstract

**Introduction:**

Programmatic assessment was introduced as an approach to design assessment programmes with the aim to simultaneously optimize the decision-making and learning function of assessment. An integrative review was conducted to review and synthesize results from studies investigating programmatic assessment in health care professions education in practice.

**Methods:**

The authors systematically searched PubMed, Web of Science, and ERIC to identify studies published since 2005 that reported empirical data on programmatic assessment. Characteristics of the included studies were extracted and synthesized, using descriptive statistics and thematic analysis.

**Results:**

Twenty-seven studies were included, which used quantitative methods (*n* = 10), qualitative methods (*n* = 12) or mixed methods (*n* = 5). Most studies were conducted in clinical settings (77.8%). Programmatic assessment was found to enable meaningful triangulation for robust decision-making and used as a catalyst for learning. However, several problems were identified, including overload in assessment information and the associated workload, counterproductive impact of using strict requirements and summative signals, lack of a shared understanding of the nature and purpose of programmatic assessment, and lack of supportive interpersonal relationships. Thematic analysis revealed that the success and challenges of programmatic assessment were best understood by the interplay between quantity and quality of assessment information, and the influence of social and personal aspects on assessment perceptions.

**Conclusion:**

Although some of the evidence may seem compelling to support the effectiveness of programmatic assessment in practice, tensions will emerge when simultaneously stimulating the development of competencies and assessing its result. The identified factors and inferred strategies provide guidance for navigating these tensions.

**Electronic supplementary material:**

The online version of this article (10.1007/s40037-020-00625-w) contains supplementary material, which is available to authorized users.

## Introduction

Programmatic assessment is a specific approach to designing assessment programmes with the aim to simultaneously optimize the decision-making and learning function of assessment. Although all educational programmes using a variety of assessments can be identified as assessment or evaluation programmes, programmatic assessment refers to a systematic approach to purposefully integrate a series of individual, dual-purpose measurements [1]. Two principles are considered unique and distinctive for programmatic assessment: the principle of proportionality and the principle of meaningful triangulation [2], both defined further below.

Compared to the traditional formative or summative purpose of assessment, programmatic assessment implies an assessment continuum which ranges from individual low-stakes assessments to high-stakes decisions [3]. This means that all types of formal and informal assessments and feedback are low-stakes and focused on providing progress information to support learning in all competency domains. In programmatic assessment, information about learners’ competence and progress is purposively and continually collected and analysed. There is an emphasis on longitudinal assessment of the learner, which creates a continual flow of information. High-stakes decisions are based on expert judgements of students’ progress, which requires interpretation of the combination of results of a variety of assessment methods [1, 3, 4]. The principle of proportionality refers to the association between the stakes of an assessment decision and the richness of the information on which that decision is based. Furthermore, in contrast to the traditional one-instrument-to-one-competency domain assessment programme, all competency domains are informed by various information sources [1, 4], which is referred to as the principle of meaningful triangulation.

In summary, programmatic assessment involves the careful selecting and combining of a variety of assessment methods and activities, which are meaningfully embedded in educational design, in order to promote learning as well as to obtain ‘a whole picture’ of learners’ performance to inform high-stakes decisions [1, 3, 4].

There has been great interest in programmatic assessment, as it theoretically aligns with the goals and curricula of competency-based medical education [5, 6]. Programmes ranging from undergraduate to postgraduate medical training are rapidly implementing the approach [7, 8]. However, few efforts have been undertaken to examine the current state of research on programmatic assessment in practice. In this integrative review, we synthesize the available research to understand if and how programmatic assessment supports learning as well as decision-making, in order to inform the development of theory and to guide future research and implementations.

## Methods

We conducted an integrative review of the literature. In this approach, literature on a topic is reviewed using a systematic process in order to synthesize data from studies that have used a variety of methodologies [9]. An integrative review offers a flexible approach in developing a rich and detailed interpretation to gain a comprehensive representation and understanding of programmatic assessment. This process is systematic but subjective by nature [10]. Therefore, our research team represented a diversity of backgrounds and expertise, ensuring robust and critical interpretation of the included studies. SS is a PhD candidate focusing on the educational consequences of assessment. LM is a researcher and information scientist with expertise in conducting knowledge syntheses. CvdV has extensive expertise in developing and investigating programmatic assessment. SH is a program director and experienced in implementing curricular innovations in health care professions education. ED and JvT are experienced researchers in assessment and teacher education, respectively. Our approach and findings are reported in accordance with the STORIES statement [11].

### Search strategy

SS and LM co-designed the search strategies for PubMed, Web of Science, and ERIC. Combinations of keywords and controlled vocabulary terms were optimized for each database (see Appendix I in the Electronic Supplementary Material for the complete search strategies). Initial searches were conducted on 9 July 2019. Searches were limited to citations published from 2005 to the present, as the seminal paper proposing a programmatic approach to assessment was published in 2005 [1]. A follow-up search was conducted on 8 December 2019 to assess for new, relevant articles. To ensure comprehensiveness of the approach, bibliographies of included studies were manually hand searched for additional relevant cited and citing studies.

### Eligibility criteria

Two reviewers (SS and a research assistant) independently screened all titles and abstracts. Publications were included when the implementation of programmatic assessment followed the principles of meaningful triangulation and of proportionality as described in the Introduction. To be included, studies had to contain evidence of the impact of programmatic assessment in practice by collecting and analysing empirical data; however, at this point no judgement was made regarding to the robustness of the data. Studies were excluded if they were not in English, published before 2005, did not include a health care professions population, or focused solely on the development of programmatic assessment or describing the local implementation process. Review articles, commentaries, and letters were excluded.

### Data extraction and analysis

We developed a data extraction form, which was operationalized in Microsoft Excel. Descriptive and citation information was extracted, including but not limited to: study setting, study aim, study design, study population, data collection methods, and summary of key findings. We characterized the implementation of programmatic assessment using the principles of meaningful triangulation and of proportionality as described in the Introduction. Using Kirkpatrick’s hierarchy [12, 13], we classified study outcomes on four levels: satisfaction/perception, learning outcomes, performance improvement, and patient/health outcomes. We summarized the data extraction results using descriptive statistics. To supplement these findings, we thematically analysed [14] the results of each study to identify themes in the key findings and mechanisms that enabled or hindered programmatic assessment to operate in practice. Each study was independently analysed and coded using an open coding strategy. SS analysed and coded each study in conjunction with one other independent reviewer (ED, LM, SH, CvdV, or JvT). The two co-investigators met after extracting data in each subset to review the coding process and define and agree on themes (i.e. patterns within the data) emerging from the process. In a process of constant comparison, SS collated all proposed themes, which the research team collectively discussed to create a comprehensive and shared understanding of each theme. Disagreements were discussed and resolved by the whole research team to either reach consensus or further refine the description of the proposed theme. All authors reviewed and agreed on the themes arising from the data analysis.

## Results

Based on our inclusion and eligibility criteria, 27 publications [15–41] were analysed (see Appendix II in the Electronic Supplementary Material for the literature search and selection process). We first present the general state of the literature, which is summarized in Tab. 1, and then the identified themes. The complete overview of studies is available in Appendix III of the Electronic Supplementary Material.

**Table 1:** Summary of study characteristics

	Characteristics	*n* (%)
Study design	Quantitative	12 (44.4%)
	Qualitative	10 (37%)
	Mixed methods	5 (18.5%)
Implementation location	The Netherlands	10 (37%)
	Canada	6 (22.2%)
	United States	3 (11.1%)
	Australia	1 (3.7%)
	United Kingdom	1 (3.7%)
	Iran	1 (3.7%)
	New Zealand	1 (3.7%)
	Multiple locations ^a^	4 (14.8%)
Setting	Clinical	21 (77.8%)
	Pre-clinical	3 (11.1%)
	Both	3 (11.1%)
Data sources ^b^	Learner perceptions	13 (36.1%)
	Teacher perceptions	11 (30.5%)
	Assessment data	12 (33.3%)
Kirkpatrick levels	Level 1	17 (62.9%)
	Level 2	3 (11.1%)
	Level 1 and Level 2	1 (3.7%)
	Level 3/Level 4	0 (0%)
	Not applicable	6 (22.2%)

^a^ ‘Multiple locations’ refers here only to some combination of the countries listed here

^b^ Multiple data sources in a single study add up to the total of 36 data sources indicated here (100%)

### General description of literature on programmatic assessment

The 27 studies used quantitative methods (*n* = 10), qualitative methods (*n* = 12) or mixed-method (*n* = 5) approaches. All used a competency-based educational framework and often noted that this was the reason for implementing programmatic assessment. Twenty-one studies were conducted in workplace settings (i.e. the clinical phase of medical education), three within a pre-clinical setting, and three within both clinical and pre-clinical settings. Thirteen studies explored learners’ perceptions (e.g. medical students, interns, residents), 12 explored teachers’ perceptions (e.g. assessors, mentors, coordinators, preceptors, supervisors), and 13 studies used assessment data (e.g. number of mini-CEX, grades, portfolio judgements, or quality evaluation of portfolio judgments). The majority of the studies originated from either Europe, specifically the Netherlands (*n* = 10), or North America (*n* = 9). Based on Kirkpatrick’s hierarchy [12, 13], we classified 17 studies at level 1 (satisfaction), three at level 2 (learning outcomes), and one study reported outcomes on both levels. No studies were observed at levels 3 (performance improvement) or 4 (patient/health outcomes).

### Thematic results

We identified three themes. In the first theme, the dual purpose of programmatic assessment, we present the synthesis of insights on the decision-making and learning function of programmatic assessment separately to provide a clearer understanding of each assessment purpose in a dual-purpose context. Data analysis revealed that the success and challenges of the dual purpose of programmatic assessment were best understood by four distinct albeit interacting factors. These are presented in the second and third theme: the interplay between quantity and quality of assessment information, and the influence of social and personal aspects on assessment perceptions.

### Theme 1: The integration of the decision-making and learning function of assessment

Fourteen studies reported that programmatic assessment generated sufficient information to enable meaningful triangulation and high-stakes, robust decision-making [15, 16, 18, 19, 24, 25, 29–32, 37–40]. Arguments for this conclusion involved findings such as high levels of assessors’ agreement [24, 30, 32, 37]; the perceived fairness or acceptability by learners and teachers [23, 24, 30]; satisfactory reliability estimates [15, 23–25, 29, 31, 37]; the coherent nature of the program that ensured all competencies were considered [17, 18, 21, 22, 28–30, 34, 38]; and early detection of struggling learners [15, 18, 22, 24, 32, 38], specifically of problematic progression on the domains of professionalism and communication, which went undetected prior to implementation [15, 24]. Furthermore, some assessors reported that the emphasis on learners’ self-assessment enabled insight into learners’ understanding of feedback and reflective skills as well as allowing triangulation of assessment information [24, 25, 30, 32]. Although the introduction of promotion or clinical competency committees was found to enable or improve the high-stakes decision-making [19, 30, 40], learners thought their mentors would be more credible in making this decision than such committees [16] and supervisors often opted to rely on their own observations to inform their high-stakes decisions [35]. Making a high-stakes decision depended on the quality of the available information [19, 35, 40], and this quality was often found to be poor or even problematic [17, 20–22, 27, 34, 35, 40].

Fifteen studies concluded that programmatic assessment was beneficial to and could be used as a catalyst for learning [16, 17, 20, 22, 23, 25, 27–29, 31, 33, 34, 38, 40, 41]. This conclusion was based on the findings that programmatic assessment supported learners’ self-assessment and development as lifelong learners [16, 20, 21, 24, 25, 27–30, 32, 38, 40], increased learners’ ownership [21, 28, 30, 34], allowed for targeted areas of improvement [16, 20, 22, 24, 27, 28, 33, 34, 38, 40], and shifted learners’ perception to assessment information as a learning opportunity [16, 22, 27, 28, 30, 38]. Based on the analysis of assessment information, three studies concluded that the approach benefited all learners to maximize their learning, including the students that were initially falling behind [25, 31, 32]. However, assessors raised concerns that programmatic assessment could be more challenging for learners with a non-native background due to the essential role of narratives [30], and one study reported the influence of learners’ performance level on their feedback-seeking behaviour [26]. Although learners perceiving assessment as a decision moment did not necessarily mean it was not beneficial for their learning, studies also showed assessment was commonly perceived as high-stakes or hampering the learning opportunity [17, 20–22, 33]. Specifically, the use of summative signals, such as grades, numerical scales on assessment instruments, pass/fail decisions, the obligatory nature of remediation or the mandatory uptake of assessment information in a portfolio, led to problems in interpretation of the nature and purpose of programmatic assessment. Summative signals could hamper learning opportunities, lead to more competition between learners, and result in the loss of valuable information [17, 20–22, 33–35, 40].

### Theme 2: The delicate interplay between quantity and quality of assessment information

The quantity as well as the quality of assessment and use of rich and narrative feedback was essential to create learning opportunities as well as to ensure meaningful triangulation for high-stakes decision-making [17, 19–22, 27, 34, 35, 40]. The use of multiple assessments lowered the perceived stakes of individual assessments [22, 33], guided better recall of past performance and stimulated users to monitor progress over time and focus on trends [16, 17, 20, 21, 25, 27, 28, 33, 35, 38, 40], and improved identification of strengths and weaknesses to facilitate tailored learning programs [15, 16, 18, 22, 24, 27, 28, 32, 33]. The programmatic approach sparked emphasis on direct observation and increased feedback [19–22, 28, 29, 34, 38] and enhanced dialogue on performance progress [20, 22, 28, 29, 34, 40].

Although teacher and learners considered the use of multiple assessments and the resulting documentation pivotal for analysis and follow-up [17, 20–22, 27, 33, 40], adverse effects were also reported. The increased number of assessments created a heavy workload for teachers and learners [20–22, 24, 28, 29, 33, 34] and risked assessment becoming viewed as time-consuming rather than meaningful or as a mainly bureaucratic activity [20, 22, 24, 33, 38]. In workplace-based settings, programmatic assessment was reported to negatively impact clinicians’ workflow [17, 21, 28, 34]. This also impacted learners, who described a reluctance to ask for feedback due to their awareness of teachers’ workload [17, 22, 28, 33, 35]. Assessors felt disinclined to provide honest or critical feedback because they feared the impact of feedback on learners or the extra workload that was thought to follow [21, 28, 34, 39, 40]. Moreover, teachers’ willingness to provide feedback was negatively influenced when they had to commit feedback to writing, which could corrupt the content of feedback [17, 20, 21, 28, 34]. Both teachers and learners expressed a fear of ‘gaming the system’, for example by strategic case or assessor selection [20–22, 34]. Furthermore, the use of multiple assessments sometimes led to a perceived decrease in quality: teachers’ tendency to give high marks impeded the monitoring of longitudinal development [16, 17], and generic feedback or no variation in assessment information provided limited input for the analyses of learners’ strength and weaknesses [17, 18, 20, 21, 28, 34]. This perceived decrease influenced learners’ uptake of assessment as a learning opportunity [16, 17, 20, 33] and teachers opting to use their own observations to inform decision-making rather than relying on the formal system [35]. The source of feedback played an important role in perceptions of assessment quality, like teachers’ credibility [20, 22, 35, 38, 41], and although peer feedback was more easily perceived as formative [17, 20], teachers’ feedback was often favoured over feedback from peers [20, 41].

### Theme 3: The influence of social and personal aspects on assessment perceptions

We found a safe and supportive social environment to be pivotal for learning in programmatic assessment [16, 21, 30, 33, 41]. Teacher support was identified as a key condition in fostering such an environment [16, 17, 20–22, 24, 30, 32, 33, 35, 39–41]. Teachers described their role as providing guidance, support, and resources; monitoring learners’ progress; and facilitating constructive discussions [16, 30, 35, 38–40]. Their contribution to high-stakes decisions could then result in role conflicts [34, 35, 39, 40]. For some teachers, the introduction of an independent progress committee resolved these conflicts [30, 38]. However, for others, such committees introduced difficulties in accepting loss of control over final decisions or highlighted their lack of trust in the assessment system [17, 21, 30, 35, 39, 40].

To build trusting interpersonal relationships, which were deemed necessary for assessment to stimulate learning, teachers and learners required frequent interaction and time spent together [16, 17, 21, 33, 40, 41]. Eight studies highlighted the importance of learner agency in teacher–learner relationships [16, 20, 21, 30, 33, 34, 39, 41]. Although there was evidence that learners experienced more agency in programmatic assessment [28, 30], others reported lack of control and ownership, which could jeopardize the learning potential of programmatic assessment [20, 33, 38, 41].

Eight studies reported the need for a shared understanding between teachers and learners of the nature and purpose of programmatic assessment [16, 19, 21, 29, 30, 32, 38, 40]. To achieve this understanding required sufficient training and faculty support (e.g. instructions, guidelines, consensus meetings) [16, 17, 22, 29, 30, 34, 38, 40]. This shared understanding was further impacted by teachers’ and learners’ personal values and belief systems [21, 28, 34, 35, 39]; their previous assessment experience [22, 30, 33, 40]; the level of their experience with programmatic assessment [16, 17, 20–22, 28–30, 33, 34, 38, 40, 41]; and learners’ confidence level, motivation, or orientation towards learning [20, 21, 26, 28, 33, 34, 39]. Teachers and learners had to gain trust in and understanding of the system [16, 20, 22, 28, 29, 33, 38, 41]. In six studies, the implementation of programmatic assessment resulted in a shift in the assessment culture: towards one in which daily feedback was normalized and in which learning and self-reflection could thrive [19, 24, 28, 30, 34, 38].

## Discussion

We reviewed 27 studies to generate novel insights on programmatic assessment. At first glance, the studies in our review seem to paint a rather optimistic picture of programmatic assessment in practice. Studies reported that programmatic assessment generated sufficient information to enable robust decision-making and could be a catalyst for learning. However, closer inspection revealed several problems. These included an overload in assessment information and an associated excessive workload, the counterproductive impact of using strict requirements and summative signals, lack of a shared understanding of the nature and purpose of programmatic assessment, and lack of supportive interpersonal relationships. In this section, we consider our findings in a broader context of health care professions education and assessment. In Tab. 2, we offer some inferred strategies from the literature to inform future implementations of programmatic assessment.

**Table 2:** Inferred strategies from the literature to improve the value and use of programmatic assessment

Inferred strategy and exemplifying references
Build on creating a shared understanding of programmatic assessment by clearly introducing the nature and purpose, providing explanatory guidelines for individual assessments and how they are used in the system as a whole, and involving teachers and learners in the whole chain of the system [16, 19, 21, 29, 30, 32, 38, 40]
Provide teachers and learners with feedback on the quality of provided assessment information and how their input contributes to the decision-making process [17, 21, 24, 40]
Normalize daily feedback, observation, and follow-up, as well as reflection and continuous improvement [19, 21, 22, 28, 34, 38]
Be cautious with mandatory requirements, being overly bureaucratic, and the use of summative signals in the design of programmatic assessment [17, 20–22, 24, 28, 33–35, 40], but keep the approach flexible, fit for purpose and negotiable, specifically in relation to the information needs of different stakeholders and the realities of the educational context [16, 17, 20, 21, 24, 28, 33, 34, 41]
Promote learner agency and the development of life-long learner capabilities by increasing learners’ ownership over the assessment process [20, 28, 30, 34, 41]
Address learners’ and teachers’ assessment beliefs and the implications of a learner-led assessment approach [21, 28, 34, 35, 39] and provide mentorship for novices within programmatic assessment [16, 17, 20–22, 28–30, 33, 34, 38, 40]; more experienced stakeholders can help with the transformation
Invest in prolonged and trustworthy teacher–learner relationships to create a safe and supportive environment [16, 17, 21, 33, 35, 39–41]. Frameworks such as ‘The Educational Alliance’ model [44] and the R2C2 model [45] might be helpful in this respect
Organize group discussions and ensure shared decision-making; these do not only ease teachers’ individual assessment responsibilities but can also improve the assessment outcome [19, 24, 30, 32, 34, 35, 40]
Invest in credibility and trustworthiness as quality concepts for stakeholders, the process, and the system [21, 24, 34, 40]. Norcini et al. [46] offer a quality framework for assessment systems
Ensure a supportive infrastructure (i.e. available time and resources, effective technology and sufficient faculty development), while taking the realities of the educational context into account [17, 21, 28, 34, 38, 40]
Offer leadership in times of change. Cultural change takes time and, although issues should be addressed quickly, programmatic assessment will not be implemented perfectly from the start [38]

The use of multiple low-stakes assessments played a vital role in improving high-stakes decision-making and learning. Lowering the stakes of individual assessments, however, does not exempt us from ensuring that these assessments meet certain quality standards, at least not if we want individual assessments as well as the system as a whole to remain meaningful for learning and decision-making. Although there is some ‘safety in numbers’, there is a fine line between scarcity and overload in the use of multiple assessments. To ensure quality, it seemed counterproductive to enforce top-down decisions and mandatory requirements on how much or what type of assessment information should be used for learning and decision-making. This approach likely results in tick-box activities and in both learners and teachers gaming or even corrupting the system. Assessment information must serve the information needs of learners as well as teachers, and searching for a magic number of assessment tasks seems superfluous. However, when learners and teachers are able to negotiate what constitutes a meaningful evidence base for learning as well as decision-making in their own context, this allows for more ownership and engagement with a programmatic approach by both, which is a condition for its success.

Creating a safe and supportive learning environment is pivotal for unlocking the potential of programmatic assessment. In this endeavour, committing to prolonged and supportive interpersonal relationships seemed to be a requirement for both teacher and learners. In health care professions education, it is often inevitable that teachers and learners only work together for a brief period of time. This places certain limitations on building trusting teacher–learner relationships and can explain why the exchange of assessment information is ineffective.

No matter how well individual assessments or assessment programs are designed or intended, if teachers and learners do not understand or agree with their function or purpose, they will likely become trivialized. Programmatic assessment requires a transition from a teacher-directed approach to a learner-led assessment approach in which the development of expertise is emphasized. This change in roles and responsibilities for teachers as well as learners will almost inevitably cause uncertainty and involve resistance. Teachers and learners are more likely to support and invest in such a change if they subscribe to its educational value and are empowered to assume ownership of it. When it is clear how this change solves problems they might have, instead of creating new ones, teacher and learners might be more inclined to endorse this transition [42]. Their understanding and commitment to this transition can be aided by their active involvement in the entire process, from the start of the decision to implement a programmatic assessment approach to its operationalization. This requires sufficient training, instructions, guidance, and support systems. Moreover, change takes time and requires strong leadership, the importance of which should not be underestimated.

Notably, the vast majority of the included studies were conducted in the clinical setting, in which workplace-based assessment plays a major and vital role. This raises the question whether a preclinical setting in which the lower levels of Miller’s competency pyramid [43] are often more dominant in learning and assessment practice is aligned with the underlying assumptions of programmatic assessment. Perhaps the approach is more compatible and therefore appealing for assessment in the clinical setting. This is an interesting question for future research. In a similar vein, we found that the majority of included articles originated in the Netherlands. This raises potential questions about the use of programmatic assessment outside this context and provides a warrant for future researchers to conduct further investigation.

### Limitations

This review must be considered in light of its limitations. Studies that did not present their assessment programme as programmatic assessment, while still investigating the same principles, might have been missed due to the nature of our inclusion criteria. Despite our efforts to be comprehensive in our searches, it is possible that we inadvertently missed a paper even though we took steps such as hand searching the papers included to safeguard against this possibility. With respect to the holistic approach of meaningful triangulation, it is important to note that several studies in our review focused on a single assessment instrument, for example the mini-CEX [21, 22, 36], the progress test [27], or a single competence such as professionalism [25, 29]. Additionally, our search strategy yielded a number of studies describing valuable lessons drawn from the local implementation process. Though these would provide valuable lessons for others aiming to implement programmatic assessment, they were outside the scope of this review.

## Conclusion

This study adds to the literature by comprehensively collecting and reviewing studies that examined programmatic assessment in practice. Although some of the evidence in the literature may seem compelling to support the effectiveness of programmatic assessment in practice, tensions will emerge when simultaneously stimulating the development of competencies and assessing its result. The identified factors and inferred strategies provide guidance for navigating these tensions.

## Caption Electronic Supplementary Material

ESM Appendix I: Database search strategy
ESM Appendix II: Literature search and selection process *Literature search and study selection process in a review of programmatic assessment in health care professions educations published 2005 to 2019.*
ESM Appendix III: Overview studies *Descriptions of 27 included studies investigating programmatic assessment in practice in health care professions education published 2005 – 2019.*
