# Supplementary material for: Where the rubber meets the road — An integrative review of programmatic assessment in health care professions education
Source: Perspect Med Educ. 2020 Oct 21;10(1):6–13. doi: 10.1007/s40037-020-00625-w (PMC7809087; doi:10.1007/s40037-020-00625-w)
Supplement: Supplementary file 1 — ESM Appendix I: Database search strategy ESM Appendix II: Literature search and selection process Literature search and study selection process in a review of programmatic assessment in health care professions educations published 2005 to 2019. ESM Appendix III: Overview studies Descriptions of 27 included studies investigating programmatic assessment in practice in health care professions education published 2005 – 2019. [file 40037_2020_625_MOESM1_ESM.docx]

**Electronic Supplementary Material**

**Appendix I: Database search strategy**

Search conducted on July 9^th^, 2019, and rerun on December 8^th^, 2019

**PubMed**

("educational measurement"[mesh] AND programmatic[All Fields])

OR ("educational measurement"[All Fields] AND programmatic[All Fields])

OR ("assessment for learning"[All Fields])

OR ("assessment of learning"[All Fields])

OR ("programmatic assessment"[All Fields])

OR ("programmatic assessments"[All Fields])

OR ("Education, Professional"[Mesh] AND “programmatic assessment”)

OR ("Students, Health Occupations"[Mesh] AND “programmatic assessment”)

OR (((medic* OR nurs* OR dent* OR pharm*) AND education) AND “programmatic assessment”))

OR ("Education, Professional"[Mesh] AND (“assessment system” OR “system of assessment” [title/abstract]))

OR ("Students, Health Occupations"[Mesh] AND (“assessment system” OR “system of assessment” [title/abstract]))

OR (((medic* OR nurs* OR dent* OR pharm*) AND education) AND (“assessment system” OR “system of assessment” [title/abstract])))

Results: 892 citations as of 7/9/2019; 947 citation as of 12/8/2019

**ERIC**

(program* AND ( "assessment for learn*" OR "assessment of learn*")) OR ("programmatic assess*") OR ("educational measur*" AND programmatic) OR "systems of assess*"

Note: Limited to 2005:

Results: 497 citations as of 7/9/2019; 504 citations as of 12/8/2019

**Web of Science**

(program* AND ( "assessment for learn*" OR "assessment of learn*")) OR ("programmatic assess*") OR ("educational measur*" AND programmatic) OR "systems of assess*"

Note: 363 citations as of 7/9/2019; 465 citations as of 12/8/2019

Total search result: 1752 citations as 7/9/2019; 1916 citation as of 12/8/2019

**Appendix II: Literature search and selection process**

Literature search and study selection process in a review of programmatic assessment in health care professions educations, published 2005 to 2019

164 additional records identified through database searching on December 8, 2019

- 55 from PubMed
- 102 from WoS
- 7 from ERIC

2 from reference list

1,752 records identified through initial database searching on July 9, 2019

- 892 from PubMed
- 363 from WoS
- 497 from ERIC

214 records published before 2005 and 194 duplicates excluded

1,510 records screened by title and abstract

1,334 records excluded based on title and abstract review

176 full-text articles reviewed for eligibility

149 articles excluded

- 4 not health care professions education
- 75 not about programmatic assessment
- 4 had different scopes
- 51 no empirical data
- 13 unable to retrieve article
- 2 not in English

27 articles included in review of programmatic assessment in health care professions education

**Appendix III: Overview studies**

Descriptions of 27 studies investigating programmatic assessment in practice in health care professions education. Published 2005 – 2019

| **Study ^ref. no^** | **Location** | **Study design** | **Setting** | **Data source (*n*)** | **Focus of study** | **Level Kirk-patrick ^a^** |
| --- | --- | --- | --- | --- | --- | --- |
|  |  |  |  |  |  |  |
|  |  |  |  |  |  |  |
| Wilkinson et al, 2011 ^15^ | New Zealand | Quantitative | Clinical | Assessment data (3539 & 826) | To describe and evaluate system to align learning and decision-making, and likelihood of passing compared with old system | 2 |
| Driessen et al, 2012 ^16^ | The Netherlands | Mixed methods | Clinical | Learners' perceptions (670&19) | To gain more insight into learners' perceptions of the educational value and feasibility of assessment approach and robustness of final decision | 1 |
| Bok et al, 2013 ^17^ | The Netherlands | Mixed methods | Clinical | Teachers' (8) & learners' (54&18) perceptions | To explore how theory interacts with educational practice | 1 |
| Hauff et al, 2014 ^18^ | United States | Quantitative | Clinical | Assessment data (28) | To determine feasibility and results of programmatic assessment | NR |
| Chan et al, 2015 ^19^ | Canada | Quantitative | Clinical | Evaluation data (50) - CCERR tool | To describe implementation of new system, report on initial experience, and compare before and after implementation | 2 |
| Heeneman et al, 2015 ^20^ | The Netherlands | Qualitative | Preclinical | Learners' perceptions (17) | To investigate the factors of implementation that students perceive as supporting or inhibiting learning | 1 |
| Bok et al, 2016 ^21^ | The Netherlands | Qualitative | Clinical | Teachers' perceptions (14) | To explore factors influencing teachers feedback giving behavior related to mini-CEX in system | 1 |
| Castanelli et al, 2016 ^22^ | Australia & New Zealand | Qualitative | Clinical | Teachers' (18) & learners' (17) perceptions | To explore learners' and teachers’ experiences using mini-CEX in system | 1 |
| Imanipour et al, 2016 ^23^ | Iran | Quantitative | Clinical | Teachers' (8) & learners' (38) perceptions | To develop an assessment system, measure its validity, reliability, and educational impact | 1 |
| O'Brien et al, 2016 ^24^ | United States | Quantitative | Preclinical | Assessment data (156); learners' (79) perceptions | To evaluate the approach and summative portfolio judgements of portfolio review committee | 1 |
| Peeters et al, 2016 ^25^ | United States | Mixed methods | Preclinical | Assessment data (192) | To investigate triangulation of qualitative and quantitative assessments in system | 2 |
| de Jong, 2017 ^26^ | The Netherlands | Quantitative | Clinical | Assessment data (87) | To investigate if performance determines learners' feedback-seeking | NR |
| Heeneman et al, 2017 ^27^ | The Netherlands | Mixed methods | Pre-clinical & clinical | Assessment data (297); learners' perceptions (17) | To explore how a progress test embedded in programmatic approach affects test scores and learners' perceptions in comparative study | 1 & 2 |
| Li et al, 2017 ^28^ | Canada | Qualitative | Clinical | Learners' perceptions (26) | To explore and understand residents' experience perceptions of system | 1 |
| Zijlstra-Shaw et al, 2017 ^29^ | United Kingdom | Mixed methods | Clinical | Assessment data (81/66); teacher evaluations (19); learners' (9) perceptions | To determine the validity and reliability of assessment system and underlying framework | 1 |
| Bacon et al, 2018 ^30^ | Australia | Qualitative | Clinical | Teachers' (17) & learners' (29) perceptions | To explore stakeholders' experiences with a constructivist-interpretivist approach to decision-making in system | 1 |
| Bok et al, 2018 ^31^ | The Netherlands | Quantitative | Clinical | Assessment data (327974) | To investigate use of programmatic assessment to track progression within and across competencies by providing validity evidence involving generalization inferences | NR |
| Ross et al, 2018 ^32^ | Canada | Quantitative | Clinical | Assessment data (458) | To determine if programmatic assessment is associated with better identification and support for residents in difficulty compared with traditional assessment | NR |
| Schut et al, 2018 ^33^ | Canada, United States & The Netherlands | Qualitative | Pre-clinical & clinical | Learners' perceptions (26) | To explore learners’ assessment perceptions and which factors influence perceptions | 1 |
| Acai et al, 2019 ^34^ | Canada | Qualitative | Clinical | Teachers' perceptions (16) | To explore teachers' experiences of the validity of competency judgments | 1 |
| Castanelli et al, 2019 ^35^ | Australia & New Zealand | Qualitative | Clinical | Teachers' perceptions (19) | To investigate supervisors experience using WBAs for summative assessment decisions | 1 |
| de Jong et al, 2019 ^36^ | The Netherlands | Quantitative | Clinical | Assessment data (352) | To provide evidence for saturation of information in portfolio assessment and the relation between final grade and number of datapoints | NR |
| Favier et al, 2019 ^37^ | The Netherlands | Quantitative | Clinical | Assessment data (574) | To assess inter-rater reliability of portfolio assessment | NR |
| Griffiths et al, 2019 ^38^ | Canada | Qualitative | Clinical | Teachers' perceptions (12&9) | To explore preceptors' perceptions of assessment culture and their changing roles in compared start and 3 years after implementation | 1 |
| Meeuwissen et al, 2019 ^39^ | The Netherlands | Qualitative | Clinical | Teachers' perceptions (12) | To explore mentor conceptualization of dual role and role enactment in system | 1 |
| Rich et al, 2019 ^40^ | Canada | Qualitative | Clinical | Teachers' (12) & learners' perceptions (10) | To illustrate programmatic assessment model in action and identify strengths and challenges | 1 |
| Schut et al, 2019 ^41^ | Canada, United States & The Netherlands | Qualitative | Pre-clinical & clinical | Learners' perceptions (26) | To explore the student-teacher relationship and the use of low-stake assessments | 1 |

Abbreviations: mini-CEX, mini clinical examination; WBA, work-based assessment; and NR, not relevant

^a^ Kirkpatrick’s hierarchy included the following levels: level 1 (satisfaction/perception), level 2 (learning outcomes), level 3 (performance improvement), and level 4 (patient/health outcomes)
